# Review of the ant genus *Meranoplus* Smith, 1853 (Hymenoptera: Formicidae) in the Arabian Peninsula with description of a new species *M. mosalahi* sp. n. from Oman

**DOI:** 10.7717/peerj.6287

**Published:** 2019-01-18

**Authors:** Mostafa R. Sharaf, Abdulrahman S. Aldawood

**Affiliations:** Department of Plant Protection, College of Food and Agriculture Sciences, King Saud University, Riyadh, Saudi Arabia

**Keywords:** Middle East, Aberrant worker, Zoogeography, Endemic, Key, Review, Afrotropical Region, Palearctic Region, Dhofar Governorate

## Abstract

The species of *Meranoplus* Smith, 1853 of the Arabian Peninsula are reviewed based on the worker caste. Two species are recognized, keyed, and illustrated by Scanning Electron Microscope images (SEM), *Meranoplus mosalahi* and *M. pulcher*, Sharaf, 2014.* Meranoplus mosalahi* **sp. n.** is described from the Dhofar Governorate, Oman based on the worker caste. The new species belongs to the *M. magrettii*-group and closely resembles *M. pulcher* Sharaf, 2014 from the Kingdom of Saudi Arabia (KSA), from which it can be distinguished by the bicolored body, the shallowly concave anterior clypeal margin, the absence of well-developed anterior clypeal teeth, the fewer irregular longitudinal cephalic rugae, and the feeble longitudinal rugae on posterior face of petiolar node.

## Introduction

The ant genus *Meranoplus* was established by *Smith* in 1853, based on the type species *M. bicolor* (Guérin-Méneville, 1844) (=*Cryptocerus bicolor* Guérin-Méneville, 1844), by subsequent designation by Bingham (1903). *Meranoplus* is a large genus in the subfamily Myrmicinae, with 90 described species and subspecies (Bolton, 2018) distributed throughout the Old-World tropics (Fisher & Bolton, 2016), including the Afrotropical, the Oriental, the Australian, and the Malagasy regions (Brown Jr, 2000). Many of the species build nests in the ground (Boudinot & Fisher, 2013) or among plant roots (Fisher & Bolton, 2016) and the species of this genus are categorized as omnivores, or facultative or specialist granivores (Brown Jr, 2000; Andersen, 2000; Anderson, 2006).

Species of *Meranoplus* are diagnosed by the combination of the following characters in the worker caste (Bolton, 1994): antennae 9-segmented, with a 3-segmented terminal club; antennal scrobes well-developed; mandibles armed with 4–5 teeth; the promesonotum, in dorsal view, forms a remarkable wide shield structure expanded laterally and in some species posteriorly; promesonotal shield with lateral and/or posterior spines; petiole sessile; first gastral tergite forms the majority of gaster in dorsal view. The *M. magrettii*-group is identified by the following characters (Bolton, 1981): mandibles armed with 4–5 teeth; propodeum concealed when seen from above; propodeal spines well-developed; petiole cuneate in profile and without spines; postpetiole broad and nodiform.

The genus *Meranoplus* is one of the better known myrmicine genera with several revisionary contributions published on the faunas of most zoogeographical regions including the Afrotropical (Bolton, 1981), the Oriental (Schödl, 1998; Schödl, 1999), the Australian (Taylor, 1990; Taylor, 2006; Schödl, 2004; Schödl, 2007), and the Malagasy (Boudinot & Fisher, 2013) regions. *Meranoplus periyarensis* (Bharti & Akbar, 2014) was described from India based on the worker caste (Bharti & Akbar, 2014).

The first report of the genus *Meranoplus* from the Arabian Peninsula (Sharaf, Al Dhafer & Aldawood, 2014) described a new species, *M. pulcher* of the *M. magretii*-group based on the worker caste, from the southwestern mountains of the KSA. Recently, the queen caste of *M. pulcher* was discovered for the first time based on a single specimen collected from the type locality by pitfall traps (Sharaf & Aldawood, 2017). We herein review the Arabian species of the genus *Meranoplus* and report the genus for the first time from Oman based on recently collected ant specimens from the Dhofar Governorate. These specimens are described here as a new species.

## Material and Methods

### Microscopical methods

Specimens were examined with a Leica M205 C stereomicroscope with 20.5:1 zoom ratio and at a magnification of 7.8× –160× whereas measurements are taken using a M6C-9 stereomicroscope.

### Collecting methods

PT Pitfall traps.
SF Sifting trays.

Throughout the text ‘w’ indicates ‘worker’ or ‘workers’, ‘q’ for queen.

### Scanning electron microscopy

The mounted specimens were coated with platinum and imaged using a Scanning Electron Microscope, JSM-6380 LA (College of Science, King Saud University) at a resolution 3.0 nm (30KV, WD8 mm, SEI), accelerating voltage 0.5 to 30 KV, and a magnification 85 ×–400 ×.

**Figure 1:**
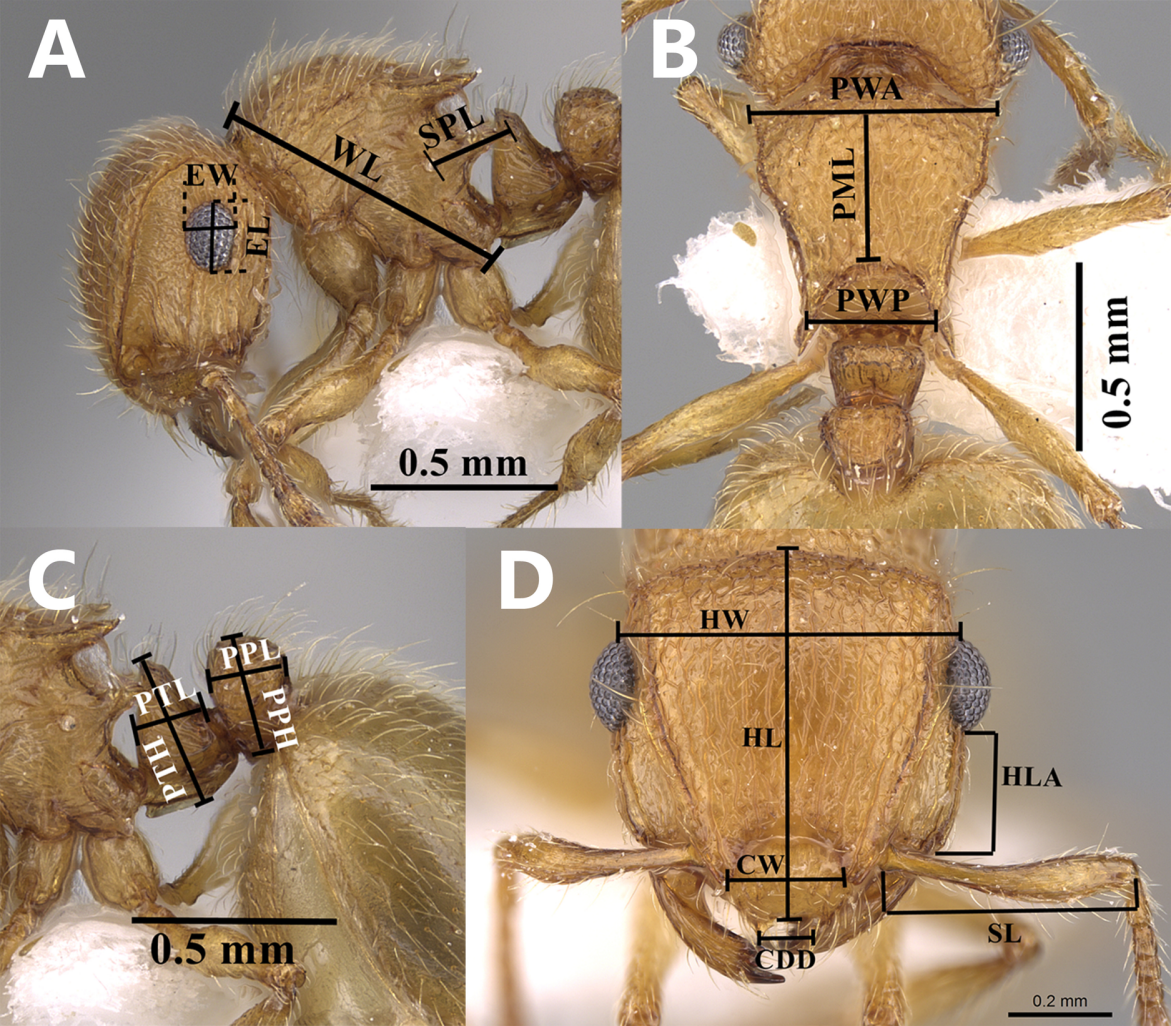
*Meranoplus pulcher.* Sharaf, illustrations of the measurements used in this paper. *Meranoplus pulcher* Sharaf, illustrations of the measurements used in this paper, (A) mesosoma in profile; (B) mesosoma in dorsal view; (C) petiole and postpetiole in profile; (D) head in full-face view, (CASENT0914336, http://www.AntWeb.org, Michele Esposito).

**Measurements and Indices.** (Figs. 1A–1D)

The following measurements and indices follow Boudinot & Fisher (2013) and Sharaf, Al Dhafer & Aldawood (2014).

ATL: *Abdominal Tergum IV Length*. Maximum length of fourth abdominal tergum measured with anterior and posterior margins in same plane of focus.
ATW: *Abdominal Tergum IV Width*. Maximum width of fourth abdominal tergum with anterior, posterior, and lateral borders in same plane of focus.
CDD: *Clypeal Denticle Distance*. Distance between clypeal denticle apices, measured in full-face view.
CW: *Clypeus Width*. Distance between apices of frontal lobes across clypeus.
EL: *Eye Length*. Maximum eye length in profile.
EW: *Eye Width*. Maximum eye width in profile.
HL: *Head Length*. Maximum length of head capsule, excluding mandibles, measured from anterior margin of clypeus to posterior margin of head, with both in same plane of focus.
HLA: *Head Length, Anterior*. Distance between anterior edges of eyes to mandible bases in full-face view.
HW: *Head Width*. Maximum width of head capsule behind eyes, in full-face view.
PML: *Promesonotum Length*. Maximum length of promesonotum from posterior spine/denticle apices to anterolateral denticle apices; all four apices in same plane of focus.
PPH: *Postpetiole Height*. Measured from sternal process base to postpetiole apex in profile.
PPL: *Postpetiole Length*. Measured from anterior to posterior inflections of postpetiole node in profile.
PTH: *Petiole Height*. Measured from petiole sternum to apex in profile.
PTL: *Petiole Length*. Measured from anterior to posterior inflections of petiole node.
PWA: *Promesonotal Width, Anterior*. Maximum width of promesonotal shield between anterolateral denticle apices in dorsal view.
PWP: *Promesonotal Width, Posterior.* Distance between posterior-most promesonotal spine or denticle apices.
SL: *Scape Length*. Maximum length of scape excluding basal constriction.
SPL: *Propodeal Spine Length*. Workers: distance from inner posterior margin of propodeal spiracle to propodeal spine apex. Queens: maximum propodeal spine length from basal inflection of spine, to spine apex.
TL: *Total length*. The outstretched body length from mandibular apex to gastral apex in profile.
WL: *Weber’s Length*. Maximum diagonal length of mesosoma from anterior inflection of pronotum to posterolateral corner of metapleuron or metapleural lobes, whichever is most distant.

**Indices**

CI: *Cephalic Index*. HW × 100/HL
CS: *Cephalic Size*. (HW+HL)/ 2× 100
EYE: *Eye Index*. 100× (EL+EW)/CS
OMI: *Ocular-Mandibular Index*. EL × 100/HLA
PMI: *Promesonotum Index 1*. PWA × 100/PML
PPI: *Postpetiole Index*. PPL × 100/PPH
PTI: *Petiole Index*. PTL × 100/PTH
PWI: *Promesonotum Index 2*. PWP × 100/PML
SEI: *Scape-Eye Index*. EL × 100/SL
SI: *Scape Index*. SL × 100/HW

**Museum abbreviations**

CASC: California Academy of Sciences collection, California Academy of Sciences, San Francisco, California, USA.
KSMA: King Saud University Museum of Arthropods, Plant Protection Department, College of Food and Agriculture Sciences, King Saud University, Riyadh, Kingdom of Saudi Arabia.
WMLC: World Museum Liverpool, Liverpool, United Kingdom.

**Nomenclatural Acts.** The electronic version of this article in Portable Document Format (PDF) will represent a published work according to the International Commission on Zoological Nomenclature (ICZN), and hence the new names contained in the electronic version are effectively published under that Code from the electronic edition alone. This published work and the nomenclatural acts it contains have been registered in ZooBank, the online registration system for the ICZN. The ZooBank LSIDs (Life Science Identifiers) can be resolved and the associated information viewed through any standard web browser by appending the LSID to the prefix http://zoobank.org/. The LSID for this publication is: urn:lsid:zoobank.org:pub: B8D5795C-FBEC-4F03-AF8C-93D299AE9BC0, and the LSID for the new species, *Meranoplus mosalahi* is urn:lsid:zoobank.org:act:75AE46DE-53B3-4948-911C-7BEA40A6D0C1 . The online version of this work is archived and available from the following digital repositories: PeerJ, PubMed Central and CLOCKSS.

**Specimens imagining**

Specimens were photographed by Michele Esposito (California Academy of Sciences, San Francisco). Digital color images of lateral and dorsal views of the entire body and full-face views of the head of each species were created using a Leica DFC450 digital camera with a Leica Z16 APO microscope and LAS (v3.8) software. These images are available online on AntWeb (http://www.AntWeb.org) and are accessible using the unique identifying specimen code.

AntWeb images included in the present work are used under a Creative Commons Attribution License mentioned on the AntWeb.org: “we encourage use of AntWeb images. In print, each image must include attribution to its photographer and “from http://www.AntWeb.org” in the figure caption. For websites, images must be clearly identified as coming from https://www.antweb.org/, with a backward link to the respective source page”.

**Recognition characters**

Throughout the work several characters are used for the separation of *Meranoplus* species and recognizing species boundaries. They are the head sculpture, the clypeal teeth and sculpture, the eyes-antennal scrobes relationships, the petiolar and postpetiolar sculpture and body colour.

## Results

### Key to the Arabian *Meranoplus* Smith, 1853

**Figure 2:**
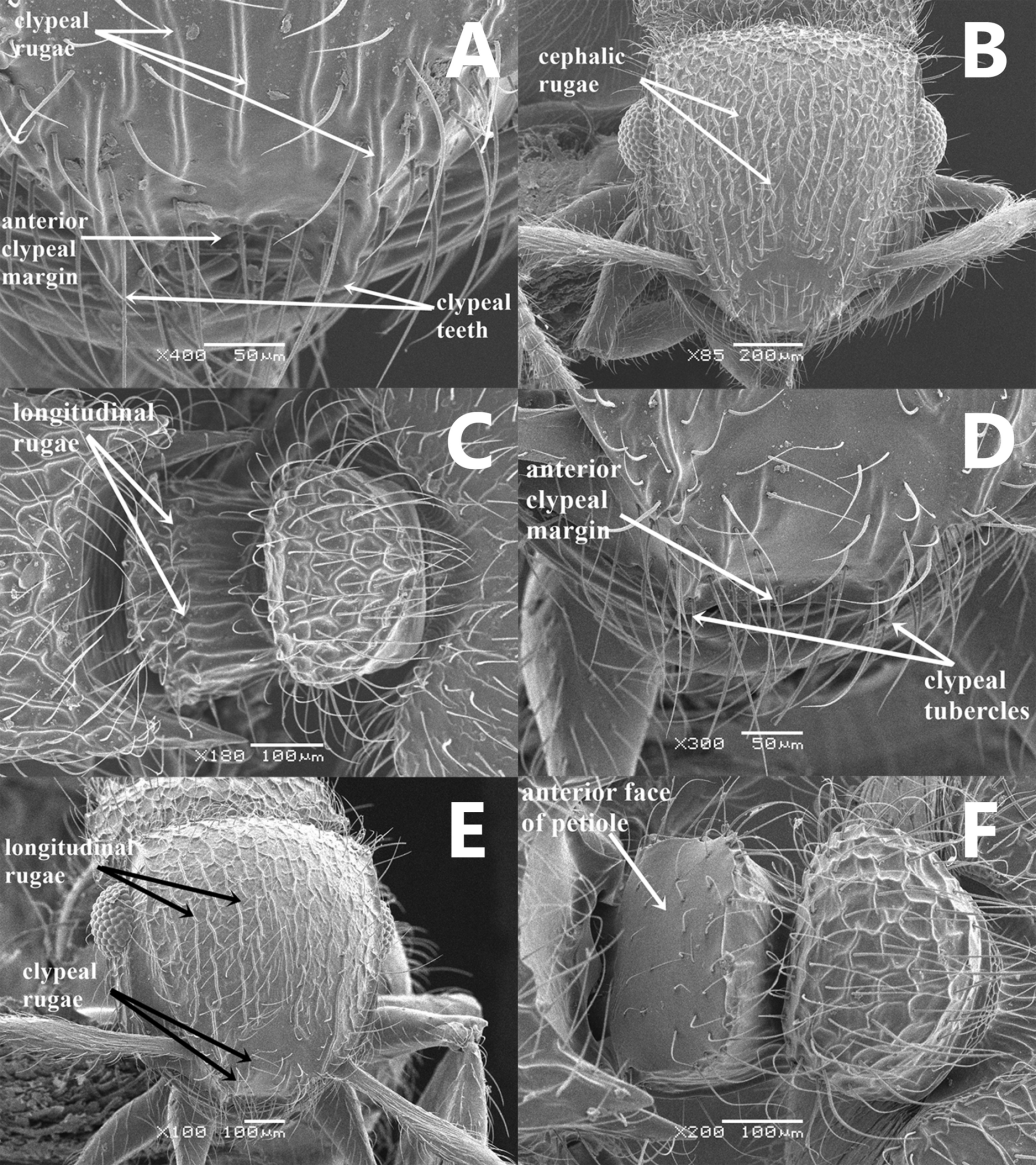
SEM images of *Meranoplus* key illustrations, (A–C) *M. pulcher* Sharaf; A, clypeus; B, Head in full-face view; C, petiole and postpetiole in dorsal view; (D–F) *M. mosalahi***sp. n.**; D, clypeus; E, Head in full-face view; F, petiole and postpetiole in dorsal view.

Anterior clypeal margin strongly concave with a single pair of well-developed blunt teeth (Fig. 2A); clypeal surface distinctly sculptured, with 3 pairs of longitudinal rugae (Fig. 2A); the inner bulge of the eye extends well into the scrobal cavity, and, in full-face view, the scrobe is broadly visible; in full-face view, cephalic dorsum to posterior level of eyes with relatively dense, continuous longitudinal rugae (about 20 rugae) (Fig. 2B); cephalic surface between rugae unsculptured; anterior face of petiolar node finely superficially punctate; posterior face of petiolar node distinctly longitudinally rugulose (Fig. 2C); uniform yellow, rarely some specimens with postpetiole and posterior margin of first gastral tergite brown (KSA)..........................................................***M. pulcher*** **Sharaf**

**-** Anterior clypeal margin shallowly concave or straight with one pair of reduced tubercles (Fig. 2D); clypeal surface unsculptured, or with two pairs of less distinct longitudinal rugae (Fig. 2E); the eye merely abuts the scrobal cavity and, in full-face view, the scrobe is narrowly visible; in full-face view, cephalic surface to posterior level of eyes with irregular interrupted longitudinal rugae (about 12 rugae) (Fig. 2E); cephalic surface with distinct fine ground sculpture between rugae; anterior face of petiolar node smooth (Fig. 2F); posterior face of petiolar node feebly sculptured with about five longitudinal rugae; distinctly bicolored, head, and gaster brown, antennae, mesosoma, petiole and postpetiole light brown, legs yellow (Oman)..............................................................***M. mosalahi*** **sp. n.**

### *Meranoplus mosalahi* Sharaf sp. n.

**Holotype worker.** Oman: Dhofar: Dhalkout, 16.70703°N, 53.25068°E, 34 m, 19.xi.2017, SF, (M. R. Sharaf), CASENT0845901, [KSMA].

**Paratype workers.** 12 w, same data as the holotype, 1 aberrant worker with reduced postpetiole, KSMA; 1 w, [WMLC], 1 w, CASENT0922861, [CASC]; Dhofar: Agdaroot, 17.088833°N, 54.442°E, 18.xi.2017, SW, (A. Mostafa), (3 w), [KSMA].

**Diagnosis.** *Meranoplus mosalahi* sp. n. can be diagnosed by the following characters: Anterior clypeal margin shallowly concave or straight with one pair of reduced tubercles; clypeal surface smooth, or with two pairs of indistinct longitudinal rugae; the eye merely abuts the scrobal cavity and, in full-face view, the scrobe is narrowly visible; cephalic surface to posterior level of eyes with irregular interrupted longitudinal rugae (about 12 rugae); ground surface between rugae finely punctate; anterior face of petiolar node smooth; posterior face feebly sculptured with about five longitudinal rugae; bicolored species with head, and gaster brown, antennae, mesosoma, petiole and postpetiole light brown, legs yellow.

**Holotype worker.**

Measurements. ATL1.10;ATW0.87;CDD0.20;CW0.27;EL0.17;EW0.12;HL0.65; HLA0.30;HW0.62;PML0.65;PPH0.25;PPL0.20;PTH0.30;PTL0.20;PWA0.65;PWP0.42; SL0.60;SPL0.20;TL3.12;WL0.80. Indices. CI95;CS0.63;EYE46;OMI57;PMI100;PPI80; PTI67;PWI65;SEI28;SI 97.

**Paratype workers.** Measurements. ATL1.03–1.37;ATW0.87–1.37;CDD0.12–0.25;CW0.22–0.30;EL0.17–0.27;EW0.12–0.17;HL0.62–0.82;HLA0.25–0.30;HW0.62–0.75;PML 0.57–0.80;PPH0.22–0.32;PPL0.12–0.25;PTH0.25–0.42;PTL0.12–0.20;PWA0.62–0.82;PWP0.30–0.50;SL0.50–0.62;SPL0.20–0.30;TL2.92–3.85;WL0.75–0.87. Indices. CI [85–108];CS0.63–0.78;EYE41–61;OMI57–108;PMI89–132;PPI50–100;PTI32-80;PWI40–74;SEI27–45;SI73–100 (*n* = 15).

**Figure 3:**
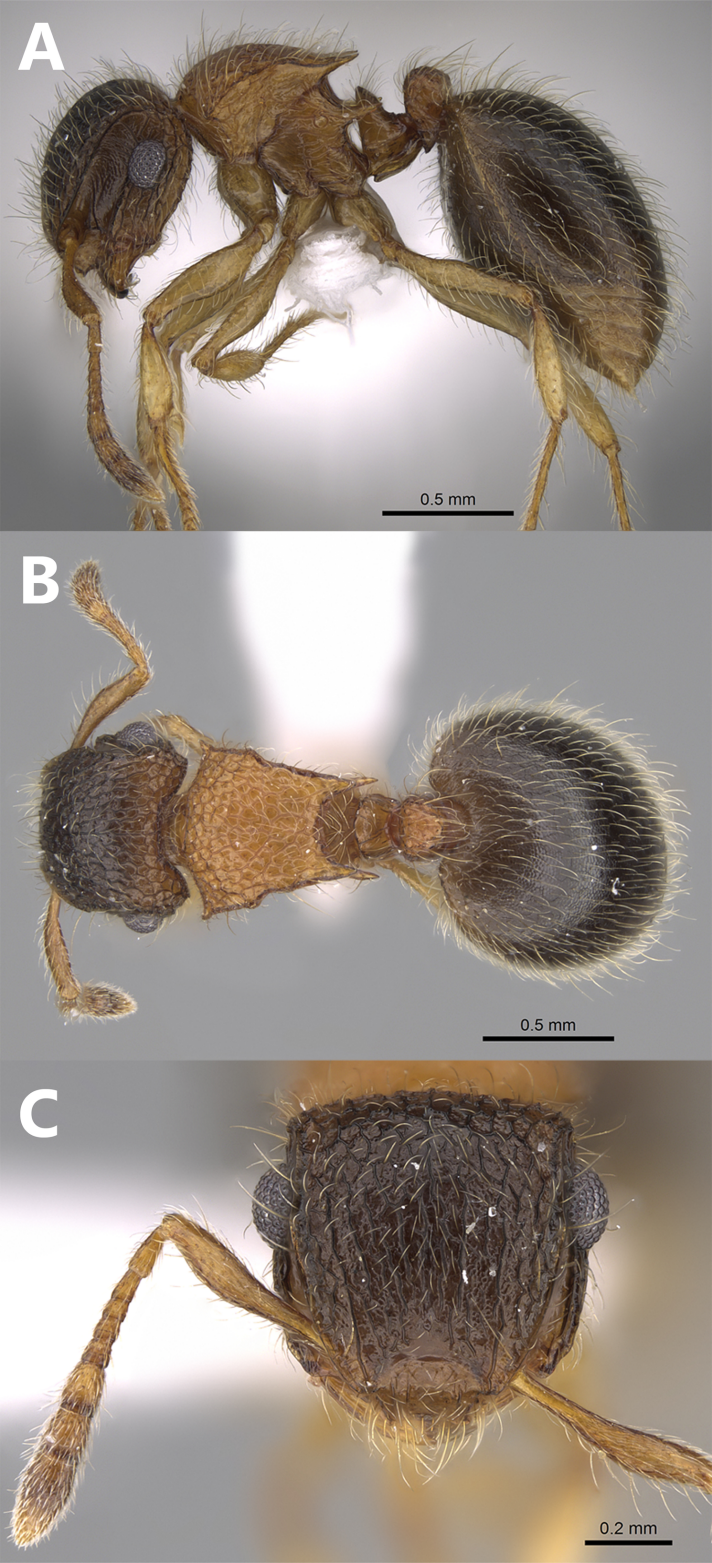
*Meranoplus mosalahi***sp. n.**, paratype worker, (A) body in profile; (B) body in dorsal view; (C) head in full-face view, (CASENT0922861, http://www.AntWeb.org, Michele Esposito).

**Description.**

**Worker** (Figs. 3A–3C). **Head.** Head slightly longer than broad with feebly convex sides and posterior margin; anterior clypeal margin shallowly concave or straight with one pair of short and blunt tubercles; clypeal surface smooth, or with two pairs of indistinct longitudinal rugae; the eye merely abuts the scrobal cavity and, in full-face view, the scrobe is narrowly visible; mandibles armed with four teeth; eyes relatively large (EYE 41–61) with about 12 ommatidia in the longest row; scapes when laid back from their insertions just reach posterior margin of eyes; scrobal carinae well-developed. **Mesosoma.** Anterior pronotal corners armed with a pair of short acute teeth seen from dorsal view; promesonotal shield distinctly broader than long (PMI 64–132) widening behind pronotum; promesonotal suture absent; posterior corners of mesonotum armed with a pair of sharp spines; posterior mesonotal margin between spines strongly concave and without secondary armament; propodeal spines long and sharp originating at level of propodeal spiracles and curved upwards; propodeal lobes well-developed. **Petiole.** Cuneate in profile, sessile, with a feebly convex anterior margin and a straight posterior margin and acute dorsum; petiolar and postpetiolar anteroventral processes well-developed. **Postpetiole.** Nodiform, distinctly higher than long in profile. **Sculpture.** Mandibles longitudinally striated; clypeus with three feebly distinct clypeal carinae; cephalic surface to posterior level of eyes with irregular interrupted longitudinal rugae (about 12 rugae), ground surface between rugae finely punctate; cephalic surface from midline of eyes to posterior margin of head distinctly areolate-rugulose or with numerous cross-meshes; antennal scrobes in front of eyes finely transversely rugulose; promesonotal shield reticulate-rugose; postpetiolar node areolate-rugose; anterior face of petiolar node smooth, sides transversally rugulose; posterior face of petiolar node feebly sculptured with about five superficial longitudinal irregular rugae; first gastral tergite finely and densely shagreened. **Pilosity.** All body surface covered with fine, pale, profuse hairs. **Color.** Distinctly bicolored, head, and gaster brown, antennae, mesosoma, petiole and postpetiole light brown, legs yellow.

**Figure 4:**
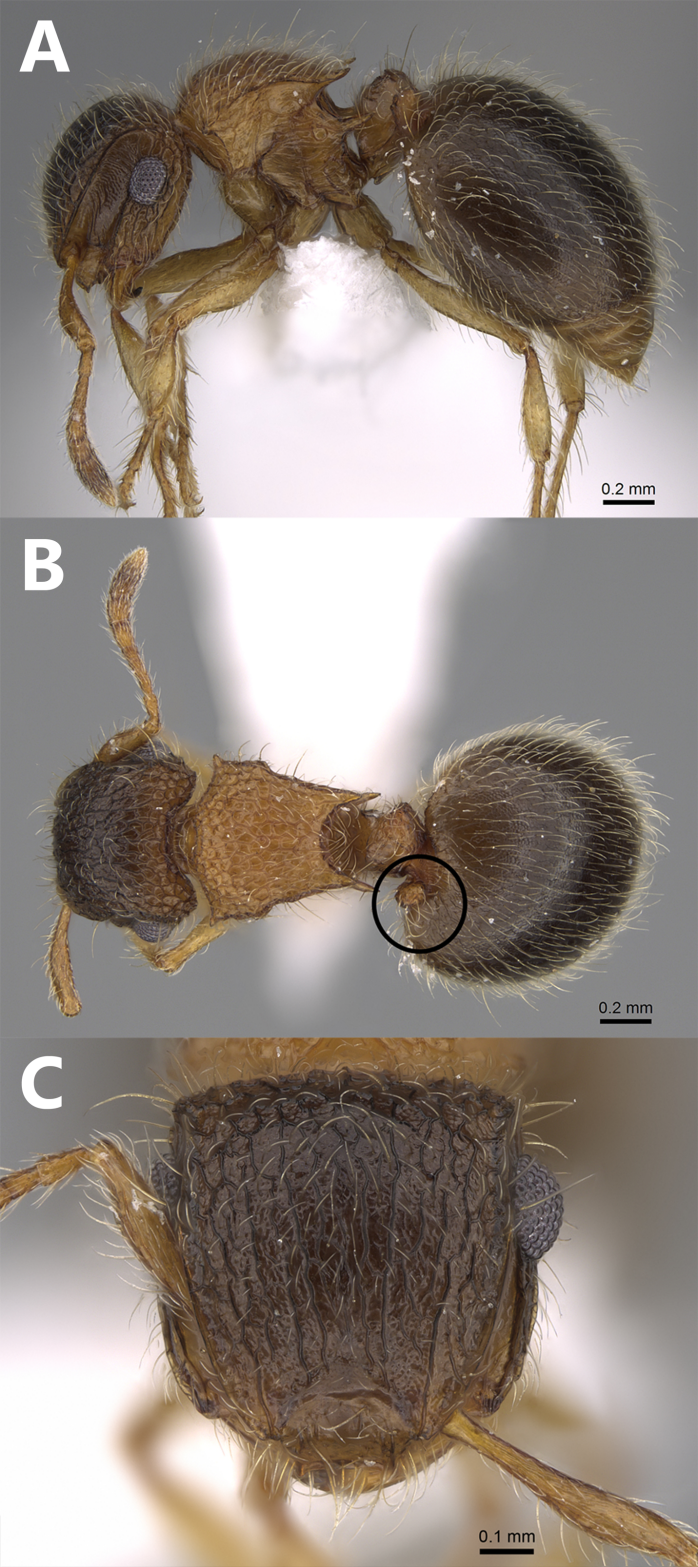
*Meranoplus mosalahi***sp. n.**, Aberrant paratype worker, (A) body in profile; (B) body in dorsal view; (C) head in full-face view, (CASENT0922862, http://www.AntWeb.org, Michele Esposito).

**Aberrant worker** (Figs. 4A–4C). A single worker was collected from the type locality with a reduced postpetiole in the form of a small bud attached to the anterior part of the first gastral tergite.

**Queen and male.** Unknown.

**Etymology.** We dedicate this species to Mohammed Salah (Mo Salah), the Egyptian professional soccer player of the English club Liverpool and the Egyptian national team.

**Remarks**

*Meranoplus mosalahi* sp. n. is a member of the *M. magrettii*-group as defined by Bolton (1981) for the Afrotropical fauna and the workers described above from Oman, could not be successfully determined using the keys of Bolton (1981) for the Afrotropical *Meranoplus* fauna. The new species is most similar to the sole known Arabian species, *M. pulcher,* especially in relative size, the well-developed anterior, posterior promesonotal and propodeal spines, the sculpture of the promesonotal shield, and the petiole and postpetiole profiles. Moreover, *M. mosalahi* can be easily distinguished by the bicolored body, the shallowly concave or straight anterior clypeal margin, the comparatively reduced anterior clypeal teeth, the weakly sculptured clypeal surface, the fewer irregular interrupted longitudinal cephalic rugae (12), and the smooth anterior face of the petiolar node. *Meranoplus pulcher* has a uniformly yellow body, rarely some specimens with postpetiole and posterior margin of first gastral tergite brown, a strongly concave anterior clypeal margin with a well-developed pair of clypeal teeth, dense longitudinal continuous cephalic rugae (20), and a finely punctate anterior face of petiolar node.

Superficially, *M. mosalahi* is similar also to *M. magrettii* André, 1884 from Sudan but the new species can be separated by the distinctly bicolored body and the strongly concave posterior margin of the promesonotal shield seen in dorsal view, which makes the posterior spines more acute, whereas *M. magrettii* has a uniform yellow to yellow-brown body, and the posterior margin of the promesonotal shield is feebly concave in dorsal view which makes the posterior spines short and blunt.

**Figure 5:**
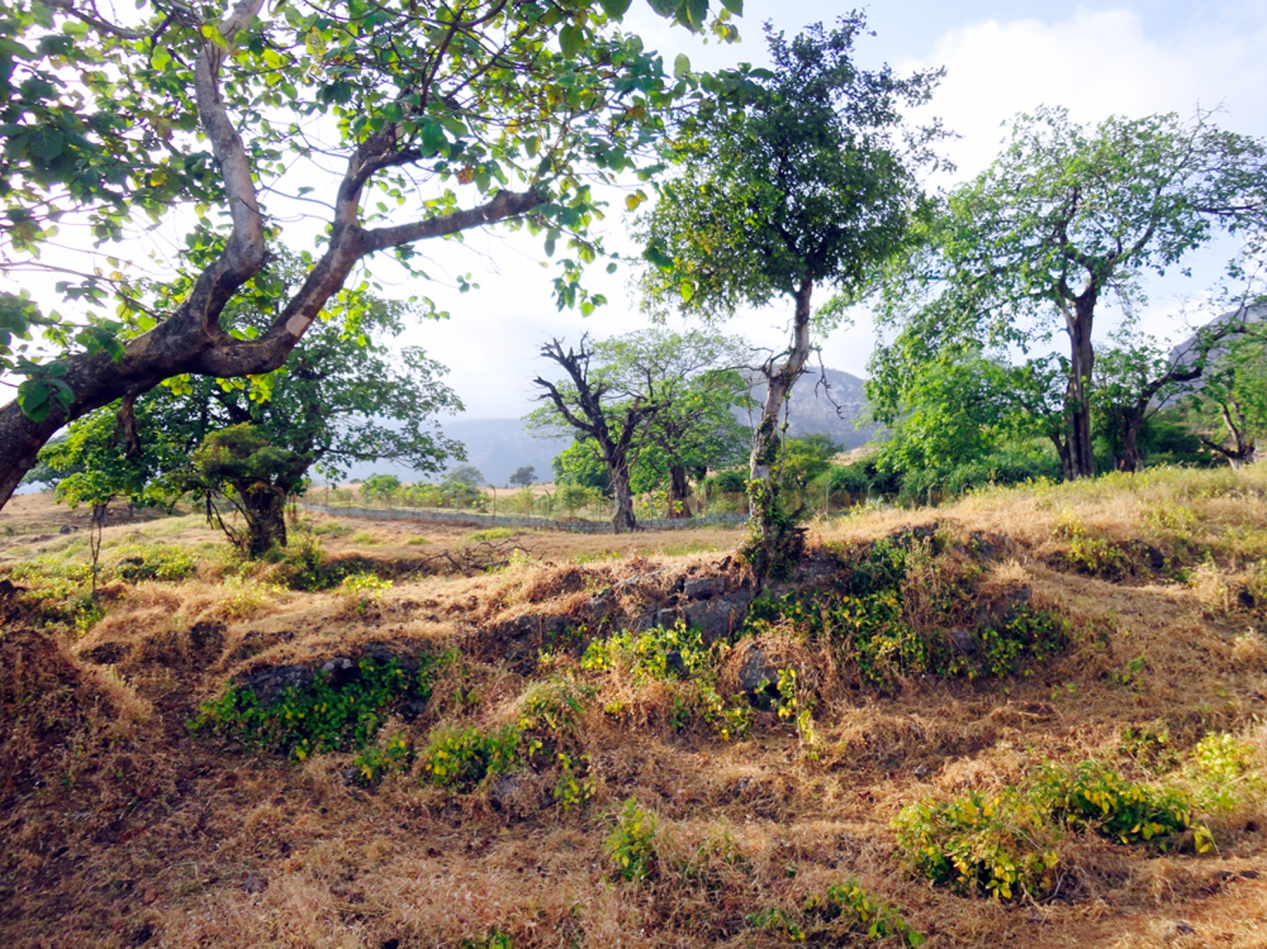
Type locality of *M. mosalahi***sp. n.**, (M. Sharaf photo).

**Ecological and biological notes.** The type locality (Fig. 5) of the new species is a shaded area with ample small shrubs and grasses. Most of the type series were relatively slow moving and were foraging on the ground where the soil was moderately humid. Some workers were collected by sweeping net.

**Geographic range.** *Meranoplus* is recorded for the first time from the Arabian Peninsula by the species *M. pulcher* from the Asir Mountains, KSA (Sharaf, Al Dhafer & Aldawood, 2014). *Meranoplus mosalahi* currently is known only from Oman and represents the first record of the genus from that country.

### *Meranoplus pulcher* Sharaf, 2014

*Meranoplus pulcher* Sharaf, 2014: 4, figa. 1–11 (w.), Holotype worker, KSA, Shada Al Ala, 19°51.066′N, 41°18.037′E, 1,325 m, 23.iv.2014, PT, (Al Dhafer *et al.*), [KSMA], Afrotropic, [examined].

**Diagnosis.** *Meranoplus pulcher* is diagnosed by the following characters: anterior clypeal margin strongly concave with one pair of well-developed blunt teeth; clypeal surface distinctly sculptured, with 3 pairs of longitudinal rugae; the inner bulge of the eye extends well into the scrobal cavity, and, in full-face view, the scrobe is broadly visible; cephalic surface to posterior level of eyes with relatively dense, longitudinally continuous rugae (about 20 rugae); cephalic surface between rugae unsculptured; anterior face of the petiolar node finely superficially punctate; posterior face of the petiolar node distinctly longitudinally rugulose; color uniform yellow.

**Worker.** Measurements. ATL0.97–1.15;ATW1.02–1.22;CDD0.12–0.15;CW0.22–0.30;EL 0.17–0.22;EW0.12–0.15;HL0.77–0.87;HLA0.25–0.30;HW0.67–0.82;PML0.40–0.52;PPH0.25–0.35;PPL0.15–0.22;PTH0.30–0.42;PTL0.12–0.20;PWA0.62–0.75;PWP0.37–0.47;SL0.47–0.62;SPL0.17–0.22;TL3.20–3.70;WL0.75–0.87. Indices. CI [87–94];CS0.72–0.84;EYE38–47;OMI63-80;PMI144–155;PPI60-80;PTI40-54;PWI82–96;SEI28–43;SI67–83 (*n* = 6).

**Figure 6:**
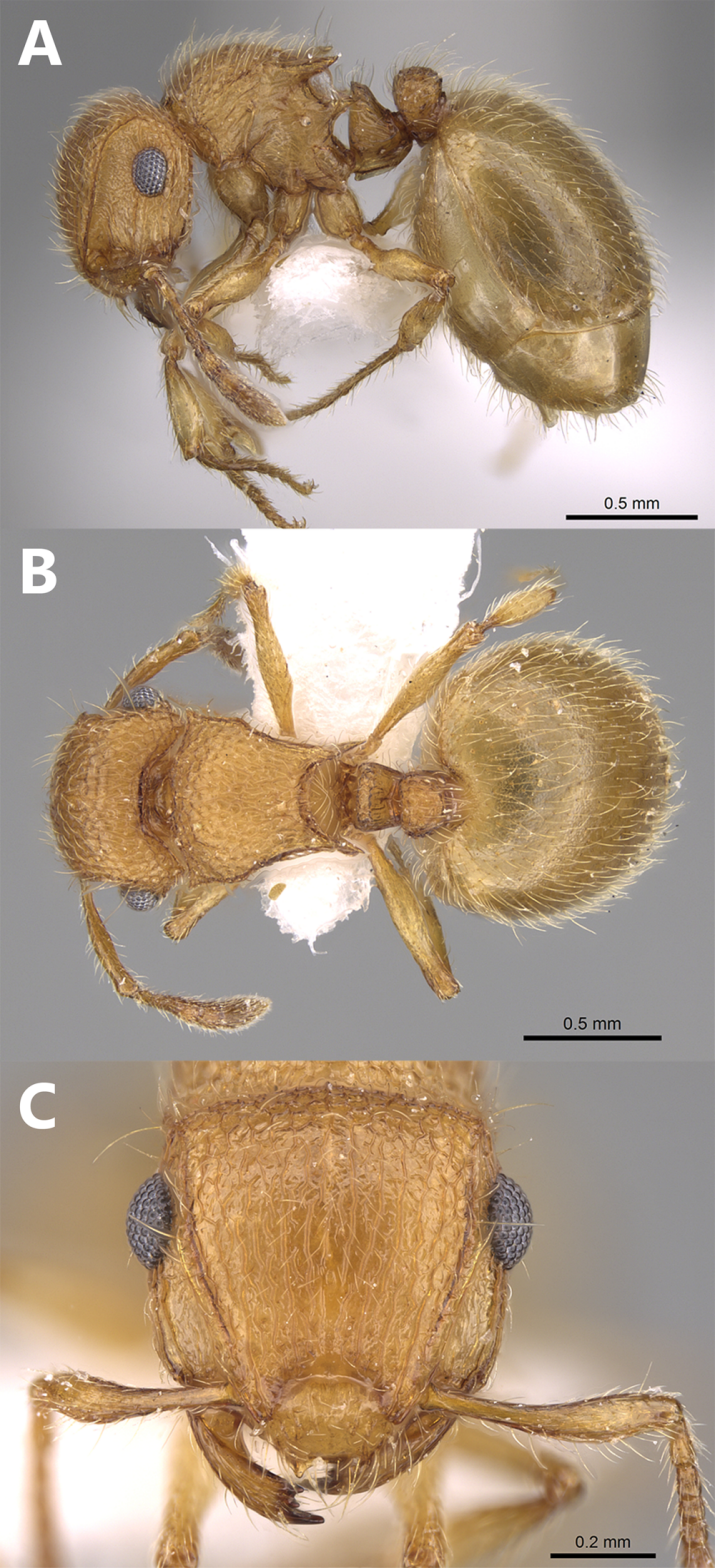
*Meranoplus pulcher* Sharaf, paratype worker, (A) body in profile; (B) body in dorsal view; (C) head in full-face view (CASENT0914336, http://www.AntWeb.org, Michele Esposito).

**Worker** (Figs. 6A–6C)**.** **Head.** Head slightly longer than broad with convex sides and straight posterior margin; anterior clypeal margin strongly concave with one pair of long and acute teeth; the inner bulge of the eye extends well into the scrobal cavity, and, in full-face view, the scrobe is broadly visible; mandibles armed with four teeth; eyes relatively large (EYE 38–47) with 12 ommatidia in the longest row; scapes when laid back from their insertions just reach posterior margin of eyes; scrobal carinae well-developed. **Mesosoma.** Anterior pronotal corners armed with a pair of short triangular teeth; promesonotal shield distinctly broader than long (PMI 144–155) widening behind pronotum; promesonotal suture absent; posterior corners of mesonotum armed with a pair of sharp spines; posterior mesonotal margin between spines strongly concave and without secondary armament; propodeal spines long and sharp originating at level of propodeal spiracles and curved upwards; propodeal lobes well-developed. **Petiole.** Cuneate in profile, sessile, with a broad anterior margin and a narrow acute dorsum; petiolar and postpetiolar anteroventral processes present. **Postpetiole.** Nodiform, higher than long in profile. **Sculpture.** Mandibles longitudinally striated; cephalic dorsum densely and finely longitudinally regularly rugulose, with about 20 rugae; cephalic surface between rugae unsculptured; posterior margin areolate-rugose or with numerous cross-meshes; promesonotal shield, anterior face of petiolar node finely superficially punctate, sides transversally rugulose; posterior face of petiolar node distinctly longitudinally rugulose (more than 10 rugae); postpetiolar node reticulate-rugulose; first gastral tergite finely and densely shagreened. **Pilosity.** Whole body surface covered with fine, pale, profuse hairs. **Color.** Uniform yellow, in some specimens, postpetiole and posterior margin of first gastral tergite brown.

**Figure 7:**
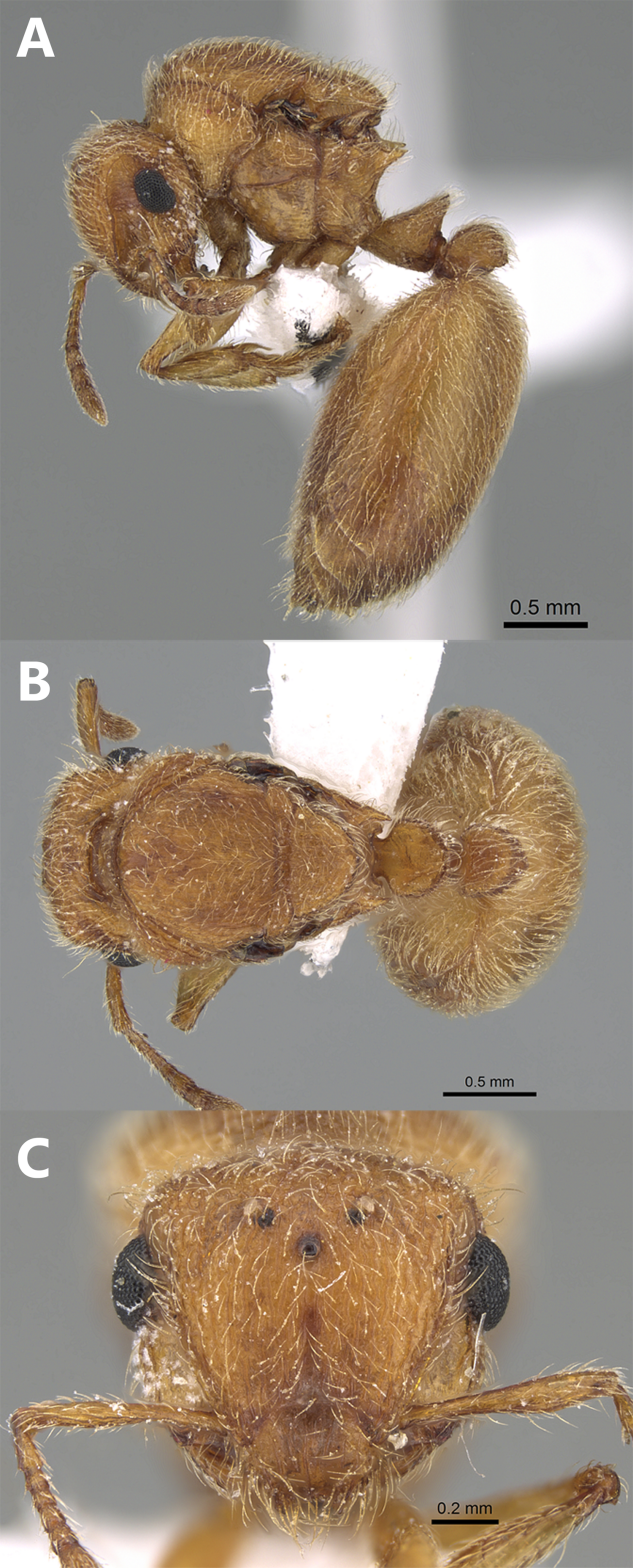
*Meranoplus pulcher* Sharaf, queen, (A) body in profile; (B) body in dorsal view; (C) head in full-face view (CASENT0922279, http://www.AntWeb.org, Michele Esposito).

**Queen** (Figs. 7A–7C).

Measurements.ATL2.05;ATW1.55;CDD0.12;CW0.37;EL0.30;EW0.17;HL0.95; HLA0.25;HW1.07;PML1.37;PPH0.42;PPL0.35;PTH0.50;PTL0.32;PWA1.15;SL0.70; SPL0.25;TL5.5;WL1.55. Indices. CI113;CS1.01;OMI120;PMI84;PPI83;PTI64;SEI43;SI65 (*n* = 1).

**Head.** Head distinctly broader than long with straight posterior margin in full-face view; eyes large (EL 0. 28 × HW); scapes short (SI 65) when laid back from their insertions just reach posterior level of eye midlength; antennal scrobes deep; anterior clypeal margin distinctly concave with prominent pair of blunt denticles. **Mesosoma.** Propodeal spines well-developed and sharply pointed. **Petiole.** Sessile, cuneate in profile, 1. 5 × higher than long in profile. **Postpetiole.** Postpetiole 1. 2 × higher than long in profile; petiole and postpetiole each without ventral processes. **Sculpture.** Cephalic surface longitudinally regularly rugulose, with interspaces between rugulae densely punctate and dull; clypeus smooth; mandibles longitudinally rugulose; three distinct oblique rugae at the middle of antennal scrobes; pronotum punctate and dull; mesosomal dorsum faintly but distinctly longitudinally rugulose; mesopleura smooth and shining; propodeal dorsum and sides transversally rugulose; anterior face of petiole unsculptured; posterior and lateral faces of petiole, entire postpetiole, and gaster densely punctate and dull. **Pilosity.** Cephalic pilosity profuse and relatively long; anterior clypeal margin and mandibles with dense long hairs; mesosomal dorsum with profuse hairs; anterior face of petiole without hairs; petiole and postpetiole dorsum with dense hairs. **Color.** Uniformly yellow.

**Habitat.** *Meranoplus pulcher* was collected near *Acacia* trees in the southwestern mountains of the KSA where soil is typically dry in an area with abundant grasses and shrubs.

**Material examined.**

**Saudi Arabia:** **Asir Province, Raydah:** 18°11.749′N, 42°23.345′E, 1614 m, 26.viii.2014, 1 paratype w., unique specimen identifier CASENT0914336, in CASC; 18°11.749′N, 42°23.345′E, 1614 m, 28.iv.2014, 1 w; 18°11.618′N, 42°23.420′E, 1772 m, 26.viii.2014, 4 w; **Al Baha Province, Shada Al Ala:** 19°50.575′N, 41°18.691′E, 1666 m, 23.viii.2014, 3 w; 19°50.411′N, 41°18.686′E, 1611 m, 23.viii.2014, 9 w; 19°50.329′N, 41°18.604′E, 1563 m, 23.vii.2014, 3 w, 1 q (CASENT 0922279) ; 19° 50.710′N, 41°18.267′E, 1474 m, 23.viii.2014, 5 w; 19°51.066′N, 41°18.037′E, 1,325 m, 23.viii.2014, 5 w, all previous material are collected by Al Dhafer et al. by using PT and deposited in KSMA.

## Discussion

The genus *Meranoplus* in the Arabian Peninsula is known now to comprise two species, *M. pulcher* and *M. mosalahi*. The former was found in the southwestern mountains of the KSA (Sharaf, Al Dhafer & Aldawood, 2014) and has not been found elsewhere despite extensive collecting efforts. Based on Sharaf, Al Dhafer & Aldawood (2014) and the available recent data, *M. pulcher* is endemic to this mountain range. This hypothesis is supported by the documented remarkable regional degree of endemism of other ant species (Collingwood, 1985; Sharaf & Aldawood, 2012; Sharaf, Aldawood & Elhawagryi, 2012; Sharaf, Aldawood & Taylor, 2012; Sharaf & Aldawood, 2013; Sharaf, Al Dhafer & Aldawood, 2018; Sharaf, Akbar & Aldawood, 2018; Sharaf et al., 2018).

*Meranoplus mosalahi* also appears to be uncommon and, perhaps, is restricted to the Dhofar Governorate and especially to the forests of Dhalkout area near the Omani-Yemeni borders. The region is known for remarkable endemism of its fauna (Arnold, 1980; Collingwood & Agosti, 1996; Soorae et al., 2013) and flora (Miller, 1994; Patzelt, 2014). We hope that future studies add more information about the unique biodiversity and the degree of endemism of the region.

### Size variation in *Meranoplus*

*Meranoplus mosalahi* exhibits distinct size variation within workers of the same nest series. This same phenomenon of size variation for the worker caste from a single nest or even between different nests have been reported for other species of *Meranoplus*. Bolton (1981) mentions the same observation for the Afrotropical species, *M. magrettii* André, 1884 and *M. peringueyi* Emery, 1886; Schödl (2007) for the Australian species, *M. unicolor* Forel, 1902; and Zryanin (2015) for the Oriental species, *M. dlusskyi* (Zryanin, 2015). Bolton (1981) attributed this size variation to be related to changes in certain morphological characters (e.g., presence or absence of sutures, length of mesonotal spines, intensity and density of sculpture, etc.).

### Teratological morphology in a *Meranoplus* worker

There are few references dealing with morphological aberration in ants but it is a documented phenomenon including all castes of fossil (Dlussky, 2009), and extant ants (Hölldobler & Wilson, 1990; Laciny et al., 2017). Aberration has been reported for males of the Malagasy species, *Malagidris alperti* (Bolton & Fisher, 2014), in which morphological deformation includes metatibiae, femora, tibiae and basitarsi of the middle and hind legs (Bolton & Fisher, 2014). The authors ascribed this deformation to a genetic aberration or a parasitoid attack during the pre-imaginal stage. The phenomenon is well studied for the queen castes of the genus *Colobopsis* Mayr, 1861, which revealed a mermithid parasitism that was examined by micro-CT imaging (Laciny et al., 2017).

Aberrant workers with a more spherical head than normal also are encountered frequently in the Neotropical species, *Wasmannia auropunctata* (Roger, 1863) (Longino & Fernandez, 2007). Among the Arabian and North African ants, however, despite more than 20 years of collecting by the senior author, the aberrant worker of *M. moslalahi* is unique. However, this phenomenon is still being investigated for the Arabian and North African species and is quite rare, since in more than 20 years of collecting by the senior author in these two regions, the aberrant worker of *M. mosalahi* sp. n. is the sole known specimen.

## Conclusion

The Arabian species of the ant genus *Meranoplus* (Smith, 1853) are reviewed, diagnosed, illustrated and keyed based on the worker caste. Two species are recognized, *M. mosalahi* **sp. n.** and *M. pulcher*, Sharaf, 2014, and a new species, *M. mosalahi* **sp. n.** is described from the Dhofar Governorate, Oman.

## Additional Information and Declarations

### Data Availability

The following information was supplied regarding data availability: 1 Holotype worker, *Meranoplus mosalahi* sp. n., King Saud University Museum of Arthropods (KSMA), King Saud University, Riyadh, Saudi Arabia (accession number: CASENT0845901). 1 Paratype worker, *Meranoplus mosalahi* sp. n., California Academy of Sciences (CASC), San Francisco, (accession number: CASENT0922861), https://www.antweb.org/bigPicture.do?name=casent0922861&shot=p&number=1
1 Paratype worker, *Meranoplus pulcher*, California Academy of Sciences (CASC), San Francisco, (accession number: CASENT0914336), https://www.antweb.org/specimenImages.do?name=casent0914336&project=allantwebants

### New Species Registration

The following information was supplied regarding the registration of a newly described species: Publication LSID: urn:lsid:zoobank.org:pub:B8D5795C-FBEC-4F03-AF8C-93D299AE9BC0 *Meranoplus mosalahi*: urn:lsid:zoobank.org:act:75AE46DE-53B3-4948-911C-7BEA40A6D0C1.
